# Saving lives with statistics

**DOI:** 10.1186/s13049-024-01256-4

**Published:** 2024-09-02

**Authors:** Jo Røislien

**Affiliations:** 1 Department of Research, The Norwegian Air Ambulance Foundation, Oslo, Norway; 2 Faculty of Health Sciences, University of Stavanger, Stavanger, Norway

**Keywords:** Statistical analysis, Trauma, Prehospital care

## Abstract

Healthcare is awash with numbers, and figuring out what knowledge these numbers might hold is worthwhile in order to improve patient care. Numbers allow for objective mathematical analysis of the information at hand, but while mathematics is objective by design, our choice of mathematical approach in a given situation is not. In prehospital and critical care, numbers stem from a wide range of different sources and situations, be it experimental setups, observational data or data registries, and what constitutes a “good” statistical analysis can be unclear. A well-crafted statistical analysis can help us see things our eyes cannot, and find patterns where our brains come short, ultimately contributing to changing clinical practice and improving patient outcome. With increasingly more advanced research questions and research designs, traditional statistical approaches are often inadequate, and being able to properly merge statistical competence with clinical knowhow is essential in order to arrive at not only correct, but also valuable and usable research results. By marrying clinical knowhow with rigorous statistical analysis we can accelerate the field of prehospital and critical care.

## Background

Statistics deals with numbers, not people, and is more concerned with group averages than individual patients. Yet, the use of statistics in medicine has been hailed as one of the most important medical developments of the last 1000 years [1].

Healthcare is full of numbers, and figuring out what knowledge these numbers hold is worthwhile in order to improve patient care. However, the human brain and our sensory apparatus is not particularly well suited for dealing with abstract numbers – particularly percentages, probabilities and other ratio concepts [2, 3] – and we need tools to help make sense of it all.

The invention of the microscope was a revolution: suddenly we could *see* things that had previously been invisible to us. With the recent increase of numerical data in society, we need new tools to see what our eyes cannot. “Mathematics is biology’s next microscope – only better,” Cohen wrote in 2004 [4].

In a world awash with numbers mathematical competence is key. By marrying clinical knowhow with rigorous statistical analysis we can accelerate the field of prehospital and critical care.

## Main

Numbers allow for objective mathematical analysis of the information at hand. However, while mathematics is objective by design – two plus two equals four regardless of where or when or by whom the calculation is performed – our choice of mathematical approach in a given situation is not. Applying mathematics for straight lines is of limited value if your problem is one of circles and curves.

In prehospital and critical care, numbers stem from a wide range of different sources and situations, and what constitutes a “good” statistical analysis can be unclear. A combination of mathematical and clinical knowhow is needed.

### Experiments

Experiments are part of the scientific bedrock. Randomized controlled trial can assess a causal association between two factors, as the rest of the world is zeroed out by design, and the accompanying numbers can be analyzed using simple statistical tests. However, with increasingly more complicated research questions and designs, even the analysis of experimental setups is not necessarily straightforward.

In a project comparing different methods for transporting trauma patients, Hyldmo et al. experimented with cadavers, meticulously measuring neck rotation and movement [5, 6]. For ethical reasons the experiment called for a non-traditional setup, and analytical results using traditional statistical tests were inconclusive. But when taking the specific structure of the experiment into account in statistical models, associations that had been hidden came into light, and the project could give concrete advice on the transportation of trauma patients.

### Observational data

When applying statistical methods to analyze our data, we simultaneously impose strict assumptions on the numbers at hand – be it the assumption of symmetrically distributed data, linear associations, or other. If these assumptions are not sufficiently correct, we hinder the numbers to communicate freely what information they actually hold.

The protein fibrinogen is vital to the body’s built-in blood stopping mechanism, and clinical guidelines state that when fibrinogen levels drop below a certain threshold mortality increases, and one should act upon it [7]. However, when looking at observational data on fibrogen levels and mortality, the numbers seem to tell a slightly different story [8]. Standard regression models assume linearity in the data. While the association between two variables is provably linear on a small enough scale [9], and linear regression thus often a suitable – and common – statistical analysis, this does not necessarily hold true on larger scales. By applying a more flexible statistical approach with fewer assumptions – applying Generalized Additive Models (GAM) rather than Generalized Linear Models (GLM) – to fibrinogen data, the critical value for what should be considered too low fibrinogen levels, is found to be substantially higher than indicated in the guidelines [8]. Applying more advanced statistical methodology directly impacts the analytical results, and the accompanying clinical conclusions.

### Data registries

More frequent use of data registries provides large amounts of healthcare data, without having to set up experiments or collect observational data from scratch. However, data registries are not designed to answer specific research questions, and results must be evaluated accordingly.

The trauma registry at Oslo University Hospital in Norway holds thousands of individual events. Plotting them on a timeline reveals a seasonal pattern [10], with more trauma admissions in summer than in winter. However, there’s not much we can *do* about seasonal changes. Seasonality just is. However, with changing seasons comes changing weather, and weather matters. Replacing the generic phenomenon “seasons” with daily factors like “hours of sunlight” and “amount of rain” [11] results in a statistical model that is not only significantly better [10], but also allows for action. Rather than planning work schedules at ERs weeks in advance, the statistical model implies that it would be more cost-efficient to ask meteorologist for estimates of sunlight and perspiration a few days ahead, calculate the expected number of trauma incidents, and staff up accordingly. More staff on sunny days, fewer when it rains.

Or – maybe not. While a statistical model with weather variables as predictors might be objectively better than mere seasonal effects, it would also result in markedly poorer quality of life for the healthcare personnel involved, having their work schedule decided by short-term weather forecasts. Statistician George Box has said that “All models are wrong, but some are useful” [12]. Even a “good” statistical analysis is not necessarily useful.

## Conclusion

Statistical analysis has two ingredients: Mathematics and context. Mathematics is often the easy part: It’s either right or wrong. The real world, however, is rarely black or white, but tends to be shades of grey, and the accompanying statistical analysis will be shades of right and shades of wrong. A well-crafted statistical analysis can help us see things our eyes cannot, and find patterns where our brains come short, ultimately contributing to changing clinical practice and improving patient outcome. Being able to properly merge statistical competence with clinical knowhow is essential in order to arrive at not only correct, but also valuable and usable research results.

Can statistics save lives? Indeed. But only when the mathematical and contextual side of statistics work together.

## Data Availability

No datasets were generated or analysed during the current study.
